# Action research improved general prerequisites for evidence-based practice

**DOI:** 10.1016/j.heliyon.2021.e06814

**Published:** 2021-04-21

**Authors:** Petronella Bjurling-Sjöberg, Ulrika Pöder, Inger Jansson, Barbro Wadensten, Lena Nordgren

**Affiliations:** a Department of Public Health and Caring Sciences, Caring Science, Uppsala University, Sweden; b Centre for Clinical Research Sörmland/Uppsala University, Sweden; c Department of Patient Safety, Region Sörmland, Sweden; d Institute of Health and Caring Sciences, University of Gothenburg, Sweden

**Keywords:** Evidence-based practice, Action research, Clinical pathway, Implementation, PARIHS

## Abstract

The present study was part of an action research project that was performed to implement a clinical pathway for patients on mechanical ventilation and simultaneously explore the implementation process in a Swedish intensive care unit. The aim of this questionnaire study was to evaluate whether an action research methodology could affect the general prerequisites for evidence-based practice (EBP). Informed by the Promoting Action on Research Implementation in Health Services (PARIHS) framework, the study included registered nurses, assistant nurses and anesthesiologists in the unit at start of the project (n = 50) and at follow-up (n = 44). Data was collected with the Evaluation Before Implementation Questionnaire and the Attitudes towards Guidelines Scale.

The results revealed that the general prerequisites for EBP in the setting improved. Compared to baseline measurements, the staff at follow-up conversed significantly more about the importance of the patients’ experiences, research utilization, context and facilitation, while changes with respect to clinical experiences were not significant. The attitudes towards guidelines were perceived as positive at baseline as well as at follow-up and did not significantly change.

Longer professional experience was associated with a slightly lower probability of perceiving that the importance of research utilization was discussed and reflected upon, while belonging to a profession with longer education was associated with a higher probability of this perception. Compared to registered nurses and assistant nurses, the anesthesiologists perceived, to a greater extent, that the importance of clinical experience was discussed and reflected upon in the setting, while there was no significant association with the length of professional experience and/or specific professions regarding the other components.

In conclusion, using action research to implement a clinical pathway methodology seems to set in motion various mechanisms that improve some but not all prerequisites that, according to the PARIHS framework, are advantageous for EBP.

## 1 Introduction

Patient care is intended to be reliable and provided according to evidence-based practice (EBP), which is based on integrated knowledge from a range of sources, including scientific research, clinical experience, contextual conditions, and patients’ preferences (Rycroft-Malone et al., 2004; Scott and McSherry, 2009). Unfortunately, current healthcare systems still fail to fully achieve this commitment, thus rendering unnecessary suffering, morbidity, mortality and healthcare costs (Panagioti et al., 2019). Hence, further efforts are needed to increase reliability and EBP in care processes (Buchert and Butler, 2016), which is an issue of high relevance for all healthcare professionals.

One of the strategies to implement EBP and increase reliability in care processes is to apply structured care methodologies, such as guidelines or clinical pathways. Clinical pathways have been increasingly used worldwide and have been acknowledged to be “the future of healthcare delivery” (p326, Buchert and Butler, 2016). A clinical pathway (also known as e.g., care pathway or critical pathway) is a complex intervention, a structured multidisciplinary care plan for a defined group of patients that translate current evidence into local contexts and coordinate roles and essential activities (Lawal et al., 2016; Vanhaecht et al., 2012). When assigned to a specific patient, the pathway should be adapted to the needs and preferences of the individual person (Vanhaecht et al., 2012). Clinical pathways are proven to enhance care quality and patient safety and optimize patient outcomes and resource utilization (Asmirajanti et al., 2018; Seys et al., 2017). However, as with all interventions, the effect of a pathway is probably related to the process of implementation, which is minimally reported in current pathway publications.

In the complex context of intensive care, delimited guidelines are common (Eldh et al., 2013; Ford and Pearse, 2012), but the more comprehensive clinical pathway methodology is infrequently applied (Bjurling-Sjöberg et al., 2014; Ford and Pearse, 2012). The few publications currently indicate that clinical pathways can increase the quality of care for specific groups of patients in intensive care units (ICUs) (Aday et al., 2013; Cooke et al., 2017). There is however a lack of knowledge on how the implementation process of a clinical pathway methodology influence general prerequisites for EBP in a setting.

### 1.1 Implementation theory

Prerequisites for EBP can be explained by the Promoting Action on Research Implementation in Health Services (PARIHS) framework (Rycroft-Malone, 2004), which is frequently cited (Bergström et al., 2020). PARIHS represents the complexities of implementing evidence into practice and proposes that success is dependent on the interplay among the components *evidence, context* and *facilitation*. Each component includes conditions that range on a continuum from low to high, with more advantageous prerequisites for EBP when all conditions are at the high end of the continuum. Despite any inconsistencies in terminology and categorization, the relevance of the proposed influential components in the PARIHS framework is also reflected in several other frameworks, models and theories (Nilsen, 2015) and is recently further refined in the integrated (i)-PARIHS framework (Harvey and Kitson, 2016).

Important characteristics of the *evidence* (the innovation/intervention that is intended to be put into practice) include, for example, the underlying knowledge source, reliability, usefulness, practicality and availability. Successful implementation is more likely if the knowledge generated from different sources is valued as relevant, is reflected upon and is discussed in the setting (Harvey and Kitson, 2016). Conditions in the *context* include culture, leadership, recipients’ characteristics and attitudes, receptiveness to change, and feedback (Nilsen and Bernhardsson, 2019)*. Facilitation* is proposed to be the active ingredient in implementation, a process of enabling people to unite to achieve a common goal. It refers to both the people in a facilitator role (internal and external facilitators) and the activities performed (implementation strategies) (Kitson and Harvey, 2016). Lately, the importance of involving the recipients (those affected by and influencing the implementation) and utilizing the knowledge of the local healthcare staff regarding improvement has been increasingly recognized (Colquhoun et al., 2017; Harvey and Kitson, 2016). However, a plethora of implementation strategies with varying degrees of complexity exists, and their effect presumably relates to how well they address the specific context.

### 1.2 Action research

Action research is a methodology that aims to promote change at the same time as scientific knowledge is produced in collaboration between recipients and researchers (Winter et al., 2001). Given the potential to also empower the participants the methodology has attracted growing interest within the broad area of social service. Several successful action research projects are reported from widespread problem areas, such as inclusive residential care for older lesbian, gay, bisexual and trans people (Trish et al., 2018), structural violence of migrant workers (Bhuyan et al., 2018) and inclusion of individuals with experience of mental illness in the training of social workers (Kaszynski et al., 2019). Additionally, collaboration between recipients and researchers in action research projects is proposed to facilitate the implementation of evidence based interventions (Munten et al., 2010; Soh et al., 2011). There is however still a gap of knowledge regarding if the action research methodology can improve the general prerequisites for EBP.

### 1.3 Aim and research questions

The present study originates from an action research project that was performed to implement a clinical pathway for patients on mechanical ventilation and simultaneously explore the implementation process (Bjurling-Sjöberg et al., 2018). The aim of the part outlined in the present paper was to evaluate whether an action research methodology could affect the general prerequisites for EBP in an ICU.

The specific research questions were as follows:

I) Are there any differences between baseline and follow-up regarding conditions in the setting in terms of: a) discussions and reflections on the importance of clinical experience, patients' experiences, research utilization, contextual factors and facilitation; b) to what degree different knowledge sources are valued as evidence, and contextual factors and facilitation promoting EBP; and c) attitudes towards guidelines?
II) Are the perceptions of the conditions affected by the length of professional experiences and/or profession?

## 2 Method

### 2.1 Design

A questionnaire study, informed by the PARIHS framework (Rycroft-Malone, 2004), was performed, utilizing data from staff at the start (baseline) and finish (follow-up) of an action research project performed to implement a clinical pathway for patients on mechanical ventilation.

### 2.2 Setting and intervention

The setting was an eleven-bed Swedish ICU for medical cardiology patients and for patients in need of general intensive care. The staff included registered nurses specialized in intensive care or anesthesiology, assistant nurses, and anesthesiologists.

The initiative to implement a clinical pathway methodology through an action research project arose from collaboration between the ICU staff and the research team. The project was managed by a local interprofessional core group. This group was responsible for the project activities and acted as internal facilitators in the implementation process. Two external facilitators from the research team supported the local group and were primarily responsible for data collection and analysis. All staff members in the ICU were, to some extent, involved in or affected by the project. Through several cycles of observing, reflecting, planning and acting, which are significant for action research (Winter et al., 2001), a clinical pathway was developed and implemented. The activities and implementation strategies included the following: scrutinizing and reflecting upon current practices and existing local guidelines; conducting external searches and reviews of clinical pathways in other ICUs and existing scientific evidence; creating drafts of guidelines to be reviewed/tested by the staff and later revised; providing repeated information in staff meetings and by e-mail; and providing interactive training and reflection on how to use the clinical pathway. The clinical pathway was for interprofessional use and had a holistic scope, including essential common concerns, goals and care activities for patients on mechanical ventilation. Further details of the action research project and the clinical pathway are provided in a previous publication (Bjurling-Sjöberg et al., 2018).

### 2.3 Sample and data collection

The sampling in the present study was consecutive and convenience and included all registered nurses, assistant nurses and anesthesiologists who worked in the setting. The baseline data collection was conducted at the beginning of the project, before the staff had been involved in any project activities. The data collection at follow-up was conducted when the clinical pathway had been implemented for approximately one year. Information about the study, together with a questionnaire and a preaddressed reply envelope, was distributed in each staff member's mail box. Two reminders were sent by e-mail.

To largely cover the components that, according to the PARIHS framework, affect the likelihood of the successful implementation of EBP (Rycroft-Malone, 2004), the Evaluation Before Implementation Questionnaire (EBIQ) and the Attitudes towards Guidelines Scale (AGS) were used.

*The EBIQ* (Bahtsevani and Idvall, 2016; Bahtsevani et al., 2007) is a 35-item self-report questionnaire that includes 8 items on demographic data followed by four sections concerning components that, based on the PARIHS framework, affect the likelihood of successful implementation. Each section starts with a question with closed response alternatives (yes, no or do not know) regarding whether respondents discuss and reflect upon the importance of the specific component in the workplace. This initial question is followed by 4–9 items on 11-point scales, with contradictory statements as anchors that illuminate conditions that are less promoting (low, 0) versus those that are more promoting (high, 10) of the implementation of EBP. The sections include the following components: *clinical experience* (5 items, Cronbach's alpha [α] = .77 in the present sample), *patient experience* (5 items, α = .84), *research utilization* (4 items, α = .78) and *context and facilitation* (9 items, α = .81).

*The AGS* (Elovainio et al., 1999) is a 14-item scale covering the factors underlying attitudes towards clinical guidelines. Each item consists of either a positive or negative (reversed) statement, answered on a seven-point scale ranging from strongly disagree (1) to strongly agree (7). In the present study, a modified version of a previous literal Swedish translation of the AGS was used. Originally, the scale includes seven subscales (Elovainio et al., 1999). In the present sample, however, Cronbach's alpha for two of the subscales was very low (impracticality α = .30; availability α = .27), and the corrected item-total correlation was low (<.3) for three of the reversed questions. An audit of the response pattern indicated translation issues for these three items. Those items were thereby excluded, and an 11-item total scale was used; this scale had satisfactory internal consistency (α = .86).

### 2.4 Data analysis

Data analyses were performed using SPSS Statistics version 22.0.0.0 (IBM, New York). The significance level was set at *p* < .05. Descriptive statistics (number [n] and distribution [%]) for demographics were calculated. To analyze differences between sample characteristics at baseline and follow-up, t-tests for equality of means (2-tailed) were used for ratio scale data (age, years in the profession and years in the current setting), and Pearson chi-square tests were used for nominal scale data (gender, profession, full/part time employment, and working shift).

To identify if conditions in the setting had changed between the start of the project and follow-up, independent sample tests were used, staff turnover precluded paired tests. The Chi-square test [*x*^2^] was used for nominal data, i.e., the four starting questions on the EBIQ. The response alternatives (yes, no, do not know) were dichotomized into yes vs. no/do not know. The Mann-Whitney U-test (2-tailed) [U] was used for ordinal data, i.e., the EBIQ subscales and the AGS 11-item total scale. In addition, each item is reported with its descriptive (median [md], interquartile range [IQR], and range [min-max]). For the EBIQ 11-point scale, a median score of five was used as an arbitrary point of comparison between leaning towards less promoting (low) or more promoting (high) conditions. For the AGS seven-point scale, a median score of four was used for leaning towards negative (disagree) or positive (agree) attitudes.

To identify whether the length of the responders' professional experience and/or profession affected their perceptions of the conditions, i.e., if they perceived that they discussed and reflected upon the importance of clinical experience, patient experience, research utilization, or context, logistic regression analyses with the enter method were performed. In the regression models (which included both datasets, i.e., baseline and follow-up), responses for the initial questions on the EBIQ (no/do not know [0] vs. yes [1]), were used as dependent variables, while ‘profession’ (with assistant nurses as the reference category) and ‘years in profession’ were used as independent variables. Missing values (n = 2–5) were excluded from the analysis. Odds ratio [OR] and 95% confidence interval [CI] were estimated.

### 2.5 Ethical considerations

The study was approved by the Uppsala Regional Ethical Review Board (2012/166) and the ICU management. The implementation of a clinical pathway and the evaluation was part of the ICU quality improvement work and was not considered to expose the patients to any risks of harm. The staff was informed that participation in the present study was voluntary. Consent was assumed as the questionnaires were returned. All data were handled to ensure confidentiality.

## 3 Results

### 3.1 Sample characteristics

The staff at the ICU consisted of a total of 60 people at baseline and 62 people at follow-up, of which 50 and 44, respectively, responded to the questionnaire. In the total sample, the response rate was 77.0% (77.4% for assistant nurses, 84.3% for registered nurses, and 55.6% for anesthesiologists). At follow-up compared to baseline, respondents were significantly younger, had fewer years in the profession, and had fewer years in the current ICU. There were no differences between groups regarding the distribution of gender, profession, level of employment, or working shift (Table 1).

**Table 1:** Characteristics of the respondents.

		Baseline		Follow-up		
		n (%)		n (%)		*p*
Gender	Female	45 (90)		36 (81.8)		
	Male	5 (10)		8 (18.2)		.252
Profession	Assistant nurse	23 (46)		18 (40.9)		
	Registered nurse	23 (46)		20 (45.5)		
	Anesthesiologist	4 (8)		6 (13.6)		.657
Level of employment	Full-time	39 (78)		39 (88.6)		
	Part-time	11 (22)		5 (11.4)		.171
Working shift	Both day and night	39 (78)		37 (84.1)		
	Day only	7 (14)		4 (9.1)		
	Night only	4 (8)		3 (6.8)		.729

		Mean (SD)	Min-max	Mean (SD)	Min-max	

Age (years)		50 (8.82)	31–65	45.7 (11.4)	24–66	.045
Years in the profession		24.4 (10.5)	3–40	18.3 (11.6)	1–40	.009
Years at present ward		15.5 (10.2)	1–36	11 (10.1)	1–37	.031

### 3.2 Perceptions of the conditions in the ICU at baseline and follow-up

Compared to baseline, a significantly larger proportion of respondents perceived at follow-up that they discussed and reflected together on the importance of *patients' experiences*, *research utilization* and *context and facilitation*. No significant differences were identified regarding *clinical experience* (Table 2). Logistic regression analyses showed that the results also remained when the potential interaction effects of profession and length of professional experience were considered, with significant differences emerging for *patients’ experiences* (OR 25.9, CI 4.11–163, *p* = .001), *research utilization* (OR 4.31, CI 1.44–12.9, *p =* .009), and *context and facilitation* (OR 5.86, CI 1.34–25.6, *p* = .019) but not for *clinical experience* (*p* = .413).

**Table 2:** Occurrence of discussions and reflections on the importance of *clinical experience*, *patients' experience*, *research utilization*, and *conditions and facilitation* in the setting, comparing respondents’ perceptions at baseline (n = 50) and follow-up (n = 44).

	Baseline		Follow-up			
	Yes	No/do not know	Yes	No/do not know		
	n (%)	n (%)	n (%)	n (%)	*X*^*2*^	*p*
Clinical experience	19 (39.6)	29 (60.4)	22 (50.0)	22 (50.0)	1.01	.315
Patients' experience	10 (22.2)	35 (77.8)	34 (77.3)	10 (22.7)	27.0	<.001
Research utilization	10 (20.4)	39 (79.6)	23 (53.5)	20 (46.5)	10.9	.001
Conditions and facilitation	19 (39.6)	29 (60.4)	29 (70.7)	12 (29.3)	8.64	.003

The respondents also scored significantly higher at follow-up than at baseline for the subscales *patients’ experiences*, *research utilization*, and *context and facilitation* but not for *clinical experience*. The item-by-item analyses showed that the scores leaned towards the higher extreme (i.e., Md > 5) on 20 items at follow-up versus ten items at baseline (Table 3).

**Table 3:** Results regarding differences between baseline and follow-up for the item-by-item analyses of the Evaluation Before Implementation Questionnaire (EBIQ)†.

EBIQ subscales and the anchor statements in the included items		Baseline (n = 50)				Follow-up (n = 44)					
Low (score 0)	High (score 10)	n	Md	IQR	Min-max	n	Md	IQR	Min-max	U	*p*
***Clinical experience***											
Clinical experience is discussed unsystematically without critical reflection	Clinical experience is discussed systematically with critical reflection	46	4	3	0–8	41	5	3	0–8		
Clinical experience of staff is not judged	Clinical experience of staff is judged	46	5	5	0–9	41	6	3	1–9		
Mutual understanding is lacking within my profession concerning the value of clinical experience	Mutual understanding exists concerning the value of clinical experience	46	6.5	4.3	1–10	41	7	3	1–9		
Clinical experience is not valued as a form of evidence	Clinical experience is valued as a form of evidence	46	5	3	0–10	40	6	3	2–10		
Clinical experience is valued as the only form of valid knowledge in decision making	Clinical experience is valued as one of several forms of valid knowledge in decision making	45	7	3.5	0–10	40	7	2.8	2–10		
**Total score** (5 items)		**46**	**5.5**	**3**	**1–9**	**41**	**6**	**3**	**1–9**	**786**	**.176**
***Patients' experience***											
Patient narratives and experience are not used	Patient narratives and experience are used	48	5.5	4	1–10	43	8	2	4–10		
Patients are not involved in the planning of care actions	Patients are involved in the planning of care actions	48	5	3.8	0–9	43	6	4	0–10		
Patients are not respected as collaborators/partners	Patients are respected as collaborators/partners	48	5	4	0–9	43	7	3	0–10		
Patients' experiences are not valued as a form of evidence	Patients' experiences are valued as a form of evidence	48	5	4	1–9	42	6.5	3	1–9		
Patient's experiences are valued as the only valid knowledge in decision making	Patient's experiences are valued as one of several forms of knowledge in decision making	47	7	3	1–10	43	7	2	1–10		
**Total score** (5 items)		**48**	**5**	**4**	**1–9**	**43**	**7**	**3**	**2–9**	**639**	**.002**
***Research utilization***											
Utilized research is poorly conceived and executed	Utilized research is well conceived and executed	43	6	3	2–8	40	7	2	4–10		
Research is not valued as a form of evidence	Research is valued as a form of evidence	43	7	3	2–10	39	8	3	5–10		
Research is valued as the only valid knowledge in decision making	Research is valued as one of several forms of valid knowledge in decision making	44	7	3	1–10	40	7	2.8	2–10		
Research is respected as certain and established knowledge	Research is respected as knowledge, but the significance must be appraised	44	5	2	1–10	40	6	3.8	1–10		
**Total score** (4 items)		**44**	**6.25**	**3**	**2.5–10**	**40**	**7**	**2**	**5–10**	**634**	**.026**
***Context and facilitation***											
The context is not receptive to change	The context is receptive to change	47	7	2	1–10	42	7	1.5	4–10		
The context is characterized by a culture that promotes a task-driven organization	The context is characterized by a culture with a holistic perspective (learning organization)	46	5.5	3.3	1–9	42	6	3	1–9		
The context is characterized by a culture that is unclear about ruling values and beliefs	The context is characterized by a culture that is clear about ruling values and beliefs	46	5	1	2–10	42	7	2	3–10		
The context is characterized by traditional (command and control) leadership	The context is characterized by transformational (visionary) leadership	47	5	3	2–10	42	6	2	2–10		
The context is characterized by a form of leadership with an autocratic and governing approach to learning, teaching, and managing	The context is characterized by a form of leadership that encourages and utilizes individual and team knowledge	47	7	3	2–10	42	7	3	2–10		
Clinical performance and economic and experience evaluations relay on a single/few methods	Multiple methods are used to evaluate performance	45	5	2	2–10	41	6	2	1–9		
Absence of feedback concerning individual, team and system performance	Presence of feedback on individual, team and system performance	46	5	4	0–9	41	6	2	2–9		
Absence of facilitators, or facilitation methods are inappropriate	Presence of facilitators, and facilitation methods are appropriate	44	4.5	4	0–10	40	5	2.8	1–8		
The function and role of existing facilitators aims at doing for others (e.g., database search)	The function and role of facilitators aims at enabling others (e.g., teaching)	43	4	3	0–9	39	5	3	0–10		
**Total score** (9 items)		**47**	**6**	**1**	**2–10**	**42**	**6**	**2**	**4–8**	**671**	**.008**

† Based on the PARIHS framework, with low scores on the 0–10 scale indicating that the prerequisites for the successful implementation of evidence-based practice are weak and high scores indicating that successful implementation is more likely to occur.

Regarding attitudes towards guidelines, the scores leaned towards positive attitudes on all 11 included items at follow-up (i.e., Md > 4) versus ten items at baseline (Table 4). However, no significant differences were identified regarding total scores.

**Table 4:** Results for the Attitudes towards Guidelines Scale (AGS) at baseline and follow-up.

Disagree – Agree (score 1–7)	Baseline (n = 50)				Follow-up (n = 44)					
	n	Md	IQR	Min-max	n	Md	IQR	Min-max	U	*p*
**AGS**										
Guidelines are useful as educational tools	50	5	1	2–7	43	6	2	3–7		
Guidelines are a convenient source of advice	50	6	1	2–7	43	6	2	4–7		
Guidelines can facilitate communication with patients and their relatives	50	5.5	1	3–7	42	5	3	1–7		
Guidelines can improve the quality of healthcare	50	6	1	4–7	43	6	1	3–7		
Guidelines are based on scientific evidence	49	5	2	3–7	43	6	2	4–7		
Guidelines are made by experts	49	4	1	1–7	43	5	2	2–7		
My professional competence is insufficient for adopting the latest guidelines†^,^‡	-	-	-	-	-	-	-	-		
Most people in my workplace have disapproving attitudes about guidelines†	49	6	2	3–7	42	6	2	3–7		
Guidelines are not valued in our organisation†	49	6	1	2–7	42	6	1	3–7		
Implementing guidelines is too expensive for us†	48	6	2	3–7	41	6	2	3–7		
Guidelines challenge the autonomy of care professionals†	50	6	2	3–7	42	6	2	4–7		
Guidelines oversimplify the practice†^,^‡	-	-	-	-	-	-	-	-		
Guidelines are difficult to find if needed†^,^‡	-	-	-	-	-	-	-	-		
I have not seen any guidelines in our ward†	50	6	2	3–7	42	7	1	2–7		
**Total score** (11 items)	**50**	**6**	**1**	**3–7**	**43**	**6**	**2**	**4–7**	**845**	**.060**

† Negatively asked question; scores reversed in the analysis.

‡ Excluded from the analysis due to a corrected item-total correlation <.3 and, if deleted, increasing the Cronbach's alpha value.

### 3.3 Associations between length of professional experiences, profession and perceptions of the conditions

No significant associations were identified between the length of professional experience and perceptions of *clinical experience* (*p* = .816), *patients’ experiences* (*p* = .616), or *context and facilitation* (*p =* .606). However, longer professional experience was significantly associated with a lower probability of perceiving that the importance of *research utilization* was discussed and reflected upon (OR 0.953, CI 0.908–1.00, *p =* .046).

The anesthesiologists perceived, to a significantly greater extent than assistant nurses (OR 13.7, CI 1.57–120, *p* = .018) and registered nurses did (OR 13.1, CI 1.51–114, *p =* .020), that *clinical experience* was discussed and reflected upon in the workplace. There were no significant differences between assistant nurses and registered nurses (*p* = .922). The anesthesiologists also perceived to a significantly greater extent than did assistant nurses (OR 74.4, CI 6.60–839, *p* < .001) and registered nurses (OR 20.1, CI 2.03–200, *p* = .010) that the importance of *research utilization* was discussed and reflected upon. Correspondingly, registered nurses, to a significantly greater extent than assistant nurses, perceived that *research utilization* was discussed and reflected upon (OR 3.70, CI 1.78–11.6, *p* = .025). There were no significant associations between profession and responses regarding *patients’ experiences* (*p* = .222) or *context and facilitation* (*p =* .280).

## 4 Discussion

### 4.1 Prerequisites for evidence-based practice

Informed by the PARIHS framework (Rycroft-Malone, 2004), this questionnaire study evaluated the general prerequisites for EBP at baseline and at one-year follow-up after an action research project was performed that included the implementation of a clinical pathway for patients on mechanical ventilation.

The application of EBP entails the utilization of integrated knowledge from a range of sources of evidence, including clinical experience, patients' experiences/preferences, scientific research, and contextual conditions (Rycroft-Malone et al., 2004; Scott and McSherry, 2009). The clinical pathway methodology aims to enhance the quality of care by promoting EBP and teamwork (Lawal et al., 2016; Vanhaecht et al., 2012). The results of the present study show that compared to baseline, a larger proportion of the staff at follow-up perceived that they discussed and reflected together on the importance of patients’ experiences, research utilization and conditions in the context. They also scored higher on the associated EBIQ subscales exploring perceptions of the conditions in the ICU, indicating more advantageous prerequisites for EBP at follow-up compared to baseline, which is consistent with findings from a grounded theory study by Bjurling-Sjöberg et al. (2018) that was conducted within the same action research project.

The most prominent result was related to how patients' experiences were regarded in the setting. At follow-up, a majority of the staff perceived that they discussed and reflected upon patients' experiences, compared to only one-fifth of the staff at baseline, and the scores on the associated EBIQ subscale indicated that the valuation of patients’ preferences had increased. Clinical pathways include standardization; however, the methodology advocates that the plan should be adapted based on individual patient needs and preferences (Vanhaecht et al., 2012). Therefore, the result may be explained by the emphasis on person-centered care in the implementation process of the clinical pathway. It may also be explained by the recent increasing national and international interest in person-centered care (Gothenburg Centre for Person-Centred Care, 2019).

Another prominent result was that at baseline, before the staff had been involved in any project activities, only one-fifth of the respondents stated that they discussed and reflected upon the importance of research utilization in their ICU. The baseline data also revealed that research was not largely perceived as requiring critical consideration. At follow-up, a majority of the respondents stated that the importance of research utilization was discussed and reflected upon. The preference to state that the importance of research utilization was discussed and reflected upon was associated with profession. Particularly, the odds ratio for the anesthesiologist, who had the highest education level, to emphasize the importance of research utilization was high compared to the others. This finding may be explained not only by their level of education but also by the work cultures of the different professions examined. Anesthesiologists commonly appraise scientific literature, while registered nurses, according to Jansson and Forsberg (2016), do not consider scientific knowledge to be as important. According to a review (Solomons and Spross, 2011), nurses are not sure of their ability to use databases and evaluate the quality of the research. In the present study, the assistant nurses had the lowest odds of stating that the importance of research utilization was discussed and reflected upon, which may be explained by the fact that they were undergraduates without knowledge of how to search for and evaluate scientific evidence. Additionally, longer professional experience was associated with a slightly lower probability of perceiving that research utilization was discussed. This finding may be explained by a perception among the more experienced members of the staff that they already know what to do. This result may also be explained by the progress in healthcare educations, including training in research and critical reflection in later curricula. Earlier studies, however, have shown that nurses tend to become less-frequent users of databases and research two years after graduation (Forsman et al., 2010).

No significant differences between baseline and follow-up were identified related to how clinical experience was regarded in the setting. This can be explained by the fact that this source of evidence not was in focus during the implementation of the clinical pathway. Clinical experience was not valued more highly than the other components were at baseline. This is in contrast to an earlier study conducted on three medical wards and one rehabilitation ward (Jansson and Forsberg, 2016), where clinical experience was the most-valued source of evidence.

Contextual conditions and facilitation, including characteristics of the recipients, culture, leadership, and feedback, are important for EBP (Nilsen and Bernhardsson, 2019). Elovainio et al. (2000) found that attitudes towards guidelines can predict utilization and that attitudes are affected by the usefulness, reliability, practicality and availability of the guidelines. These characteristics are also highlighted by other implementation researchers as important for the uptake of the intervention/innovation (Nilsen, 2015) and probably also apply to clinical pathways. In the present study, the staff (i.e., the recipients) indicated already at baseline that the attitudes towards guidelines were positive, the context was receptive to change, and the leadership was encouraging and empowering. At follow-up, there was no significant change in attitudes, but results from the EBIQ indicated that the culture in the setting had moved towards a more holistic perspective, with a learning organization, a visionary form of leadership and more distinct set of prevailing values and beliefs. Evaluation was, to a greater extent, performed through multiple rather than single methods, and feedback and facilitation comprised a greater part of the process. This is plausibly explained by participation in the action research project.

Action research is used in variety of problem areas (e.g. Trish et al., 2018; Bhuyan et al., 2018; Kaszynski et al., 2019) and is purported to be an expedient methodology to facilitate the implementation of evidence based interventions (Munten et al., 2010; Soh et al., 2011). For example, action research projects have contributed to the successful implementation of a multidisciplinary clinical pathway for patients with chest pain (Siebens et al., 2012) and an intervention to increase awareness of the intent to communicate among mechanically ventilated patients (Noguchi et al., 2019). Furthermore, as combining implementation with a research agenda may be especially attractive to ICU staff (Weinert and Mann, 2008), the strategy to implement a clinical pathway through action research in a Swedish ICU may have contributed to the improved prerequisites for EBP in the setting. The action research methodology aims to promote change at the same time that scientific knowledge is produced in collaboration between recipients and researchers (Winter et al., 2001). Consequently, it includes facilitation, which, according to the i-PARIHS framework, is purported to be the active ingredient that enables implementation (Kitson and Harvey, 2016).

In the i-PARIHS framework (Harvey and Kitson, 2016), three different facilitator roles have been identified: the novice facilitator, the experienced facilitator and the expert facilitator. In the present project, the novice facilitators consisted of an interprofessional group from the ICU. However, despite the interprofessional intention, the anesthesiologists did not participate as much as the other professionals in the implementation process, and the clinical pathway product was mainly perceived as a nursing-related matter. The two external expert facilitators, who had substantial experience implementing EBP, supported the novice facilitators in the implementation process, but the lack of experienced facilitators in the ICU may have affected the internal work. According to Kitson and Harvey (2016), there is a need for experienced facilitators with in-depth understanding and knowledge of the organization they are working in to supervise the novice facilitators. Perhaps such individuals might have contributed to an even better outcome of the project. However, being part of an action research project means that staff members are greatly involved, which in turn can lead to improved knowledge.

The low incidence of discussion and reflection on evidence from different knowledge sources at baseline further confirms the previously highlighted need to expedite the implementation of EBP (Buchert and Butler, 2016; Panagioti et al., 2019). The findings at baseline are similar to the results from orthopedic wards when the EBIQ was used to assess the prerequisites before clinical practice guideline was implemented (Bahtsevani and Idvall, 2016). Hence, the delimited discussion and reflection on the importance of different sources of evidence may have contributed to a longer and more challenging implementation process.

As seen in the results, at baseline, the staff did not substantially discuss and reflect together on the prerequisites for EBP (the PARIHS components); however, the awareness was increased at follow-up. This progress indicates increased likelihood of future improvement implementations in the ICU to be successful. The finding can also be used to further develop the areas in which members of the staff stated lower commitment.

### 4.2 Strengths and limitations

To our knowledge, this is the first study evaluating general prerequisites for EBP before and after an action research project to implement a clinical pathway, focusing on potential changes in the conditions of the setting and thus extending the evaluation beyond the effects of the specific care process. The strength of the study includes the interprofessional approach, the follow-up over time and the use of the PARIHS framework, which provides an eclectic approach to the evaluation. The results are based on the staff's perceptions of the conditions in their ICU, mainly reflecting the collective culture and attitude, with few questions assessing their individual view. Arguably, the respondents are asked to make assumptions about their colleagues' thoughts and behaviors, which may or may not be accurate. Observational studies exploring how the staff actually conversed and acted with respect to different situations might have contributed additional information. However, the combination of the EBIQ (Bahtsevani and Idvall, 2016) and the AGS (Elovainio et al., 1999) had the strength of potentially capturing typical conditions in the ICU and thereby appraising the prerequisites for EBP.

An aggravating factor that diminishes the reliability of the results is the terminology used in the questionnaires. The EBIQ is developed in Swedish (Bahtsevani and Idvall, 2016) but includes terms that some staff members perceived to be complicated. This lack of clarity may have hampered truthful answers, especially at baseline, when the respondents were not familiar with the terminology. For the AGS (Elovainio et al., 1999), a low internal consistency and an audit of the response patterns indicated translation issues for three of the items in the utilized Swedish translation. Hence, the scale could not be used as originally intended. However, exclusion of the uncertain items yielded an 11-item total scale with satisfactory internal consistency.

Other limitations also included the following: the single setting; the low number of anesthesiologists; the staff turnover, which precluded tests for dependent groups/within-subjects tests; and the lack of a control group. Clinical pathways are complex interventions, and intensive care is a complex and unpredictable context. The time between baseline and follow-up was 3.5 years. During this period, aside from the action research project, no major transformation took place in the ICU, but everyday practice inevitably included alterations beyond the control of the research team, which hampered interpretation of causality. Hence, one must exercise caution when drawing conclusions and generalizing the results to other contexts. However, despite these limitations, this study presented valuable findings that warrant further inquiry.

### 4.3 Implications for practice

To decrease unnecessary suffering, morbidity, mortality and healthcare costs, further efforts are needed to increase reliability and EBP in care processes. Although further research is needed, the present study indicates that utilizing action research to implement a clinical pathway seems to be a promising approach to improve general prerequisites for EBP in an ICU and possibly also in other contexts of healthcare where teamwork is essential.

Based on the findings of the present study, for future research, the terminology of the EBIQ and the AGS should be further developed and tailored to the target population. Additionally, the results from the questionnaires have the potential to give internal and external facilitators, quality improvement leaders and managers the opportunity to customize implementation strategies to current conditions in the setting, which is an area that merits attention in future work.

Despite the limited study scale, the findings are of international clinical interest owing the worldwide and interprofessional issue of patients not always receiving optimal care and the need for advancement in existing knowledge of facilitating EBP and reliability in care processes.

## 5 Conclusion

This study addresses the need for increased reliability and evidence base in patient care processes. Informed by the PARIHS framework, the results indicate that general prerequisites for EBP in the setting improved when the ICU staff participated in an action research project that included implementing a clinical pathway for patients on mechanical ventilation. Compared to baseline measurements, the staff at follow-up conversed significantly more about the importance of the patients’ experiences, research utilization, context and facilitation, while changes with respect to clinical experiences were not significant. The attitudes towards guidelines were perceived as positive at baseline as well as at follow-up and did not significantly change.

Longer professional experience was associated with a slightly lower probability of perceiving that the importance of research utilization was discussed and reflected upon, while belonging to a profession with longer education was associated with a higher probability of this perception. Compared to registered nurses and assistant nurses, the anesthesiologists perceived, to a greater extent, that the importance of clinical experience was discussed and reflected upon in the setting, while there was no significant association with the length of professional experience and/or specific professions regarding the other components.

In conclusion, using action research to implement a clinical pathway methodology seems to set in motion various mechanisms that improve some but not all prerequisites that, according to the PARIHS framework, are advantageous for EBP.

## Declarations

### Author contribution statement

Petronella Bjurling-Sjöberg and Lena Nordgren: Conceived and designed the experiments; Performed the experiments; Analyzed and interpreted the data; Contributed reagents, materials, analysis tools or data; Wrote the paper.

Ulrika Pöder: Conceived and designed the experiments; Analyzed and interpreted the data; Wrote the paper.

Inger Jansson and Barbro Wadensten: Conceived and designed the experiments; Wrote the paper.

### Funding statement

This work was supported by Centre for Clinical Research Sörmland, 10.13039/501100007051Uppsala University, Sweden (DLL-856531).

### Data availability statement

Data will be made available on request.

### Declaration of interests statement

The authors declare no conflict of interest.

### Additional information

No additional information is available for this paper.
